# The Complete Mitochondrial Genome of the Beet Webworm, *Spoladea recurvalis* (Lepidoptera: Crambidae) and Its Phylogenetic Implications

**DOI:** 10.1371/journal.pone.0129355

**Published:** 2015-06-19

**Authors:** Shi-Lin He, Yuan Zou, Li-Fang Zhang, Wen-Qi Ma, Xiu-Yue Zhang, Bi-Song Yue

**Affiliations:** 1 Key Laboratory of Bio-resources and Eco-environment, Ministry of Education, College of Life Sciences, Sichuan University, Chengdu, China; 2 Sichuan Key Laboratory of Conservation Biology on Endangered Wildlife, College of Life Sciences, Sichuan University, Chengdu, China; Nanjing Agricultural University, CHINA

## Abstract

The complete mitochondrial genome (mitogenome) of the beet webworm, *Spoladea recurvalis* has been sequenced. The circular genome is 15,273 bp in size, encoding 13 protein-coding genes (PCGs), two rRNA genes, and 22 tRNA genes and containing a control region with gene order and orientation identical to that of other ditrysian lepidopteran mitogenomes. The nucleotide composition of the mitogenome shows a high A+T content of 80.9%, and the AT skewness is slightly negative (-0.023). All PCGs start with the typical ATN codons, except for COX1, which may start with the CGA codon. Nine of 13 PCGs have the common stop codon TAA; however, COX1, COX2 and ND5 utilize the T nucleotide and ND4 utilizes TA nucleotides as incomplete termination codons. All tRNAs genes are folded into the typical cloverleaf structure of mitochondrial tRNAs, except for the tRNA^Ser(AGY)^ gene, in which the DHU arm fails to form a stable stem-loop structure. A total of 157 bp intergenic spacers are scattered in 17 regions. The overlapping sequences are 42 bp in total and found in eight different locations. The 329 bp AT-rich region is comprised of non-repetitive sequences, including the motif ATAG, which is followed by a 14 bp poly-T stretch, a (AT_11 _microsatellite-like repeat, which is adjacent to the motif ATTTA, and a 9 bp poly-A, which is immediately upstream from the tRNA^Met^ gene. Phylogenetic analysis, based on 13 PCGs and 13 PCGs+2 rRNAs using Bayesian inference and Maximum likelihood methods, show that the classification position of Pyraloidea is inconsistent with the traditional classification. Hesperioidea is placed within the Papilionoidea rather than as a sister group to it. The Pyraloidea is placed within the Macrolepidoptera with other superfamilies instead of the Papilionoidea.

## Introduction

The animal mitochondrial genome is a double-stranded circular DNA molecule, 14 to 20 kb in size, which encodes a conserved set of 37 genes, including 13 protein-coding genes (PCGs) plus the two ribosomal RNA (rRNA) genes and 22 transfer RNA (tRNA) genes [1,2]. Additionally, it also contains a control region, known as A+T-rich region in insects [3], including initiation sites of the transcription and replication of the mitogenome [1,4]. While the length of the A+T-rich region vary highly in that the presence of the indels and tandem duplicated elements [5]. The mitogenome is characterized by its small size, maternal inheritance, non-recombination, and rapid evolution [1,2,6]. Mitogenomes have been studied in a variety of fields, such as structural genomic [1,7], genetic resources [8], molecular evolution [9], population genetics [10], phylogeography [11], inter-ordinal and intra-ordinal relationships [12–14].

Lepidoptera (moths and butterflies) is the second largest order in the class Insecta with more than 160,000 species described around the world [15,16]. Despite the extreme taxonomic diversity in Lepidoptera, studies of lepidopteran mitogenomes have been very limited and confined to nine superfamilies: Tortricoidea, Bombycoidea, Noctuoidea, Pyraloidea, Geometroidea, Hesperioidea, Hepialoidea, Yponomeutoidea and Papilionoidea. As of 14 July 2014, 562 insect mitogenomes have been reported or deposited in GenBank. Of these mitogenomes, 151 complete or near complete mitogenomes are from the Lepidoptera, including 15 Pyraloidea species, such as *Cnaphalocrocis medinalis* [17], *Chilo suppressalis* [17], *Diatraea saccharalis* [18], *Dichocrocis punctiferalis* [19], *Ostrinia furnacalis* [20], *Ostrinia nubilalis* [20], *Scirpophaga incertulas* [21], *Elophila interruptalis* [22], *Glyphodes quadrimaculalis* [23], *Ephestia kuehniella* [24], *Maruca testulalis* [25], *Paracymoriza distinctalis* [26], *Corcyra cephalonic* (unpublished, NC_016866), *Paracymoriza prodigalis* (unpublished, NC_020094), and *Maruca vitrata* (unpublished, NC_024099). Characterization of our new mitogenome will help provide further insights into the understanding of phylogenetic evolutionary relationships in the Pyraloidea.

Taxonomically, *S*. *recurvalis* is a member of the family Crambidae, superfamily Pyraloidea. However, the number of reported mitogenome sequences in this superfamily is very limited. For the *S*. *recurvalis* mitogenome, only a partial of COX1 gene was reported [27,28]. In this study, we sequenced and described the complete mitogenome of *S*. *recurvalis* and compared its characteristics with other known lepidopteran mitogenomes. Then we reconstructed phylogenetic relationships within nine lepidopteran superfamilies using Bayesian inference (BI) and Maximum likelihood (ML) methods.

## Materials and Methods

### DNA sample extraction

Adult individuals of *S*. *recurvalis* were collected in Chengdu, China. The samples were preserved in 95% ethanol and stored at -20°C until used for DNA extraction. The whole genomic DNA was isolated from a single sample by applying phenol-chloroform protocol [18,29]. Product and quality of the DNA was assessed by electrophoresis in a 1.5% agarose gel and staining with ethidium bromide.

### PCR amplification, cloning, and sequencing

The whole mitogenome of *S*. *recurvalis* was amplified in nine overlapping fragments. All primer sequences are shown in Table 1. Primers F1F, F4F, F4R, and F6R were from Cameron and Whiting [7], primers F3R and F5F were from Simon et al. [30], primer F8F was from Bybee et al. [31], and primer F8R was from Skerratt et al. [32]. The other specific primers were designed based on the conserved nucleotide sequences of the mitogenome sequences in homologous lepidopteran species, or the mitogenome fragments that we have previously sequenced.

**Table 1:** Primer sequences used in this study.

Region	Primer pair	Primer sequence (5’→3’)	Size (kb)
s-rRNA→tRNA^Gln (Q)^	AF1F	TTTAATAATAGGGTATCTAATCCTAGTTTAT	0.86
	AF1R	GCACAATAGTTTTTGATACTTTTAGATATAGTTTG	
s-rRNA →COX1	F1F^a^	TCGTGGATTATCAATTAWTAAACAGATTCC	3.0
	F1R	TATACTTCTGGATGTCCAAAGAAT	
COX1 →tRNA^Lys (K)^	F2F	ACTCTACAAATCATAAAGATATTGG	1.9
	F2R	GTTTAAGAGACCAGTACTTG	
tRNA^Leu(UUR)^ →COX3	F3F	TATGTAATGGATTTAAACC	2.4
	F3R^b^	TCTACA AAATGTCAATATCA	
COX3 →ND4	F4F^a^	AGTAACYAAAGGRTTRCGATGAGG	4.2
	F4R^a^	YCARCCTGAGCGAATTCARGCKGG	
ND4 →CYTB	F5F^b^	GGAGCYTCAACATGAGCTTT	2.5
	F5R	ATTACACCTCCAAGTTTATTTGGAAT	
CYTB →*rrnL*	F6F	TATGTTCTTCCTTGAGGACAAATGTC	2.1
	F6R^a^	AAATTACCTTAGGGATAACAGCG	
ND1 →*rrnL*	F7F	ATCAAAAGGAGTTCGATTAGTTTC	1.4
	F7R	CACTTGTTTATCAAAAACAT	
*rrnL* →*rrnS*	F8F^c^	CACCGGTTTGAACTCAGATCA	1.7
	F8R^d^	AAACTAGGATTAGATACCC	

a. from Cameron and Whiting [7]

b. from Simon et al. [30]

c. from Bybee et al. [31]

d. from Skerratt et al. [32]

PCR amplification conditions were as follows: an initial denaturation for 2 min at 95°C, followed by 35 cycles of denaturation for 40s at 92°C, annealing for 80 s at 53–57°C (depending on primer combinations), elongation for 1–4 min (depending on putative length of the fragments) at 62°C, and a final extension step of 72°C for 10 min. All PCR amplifications applied Takara LA Taq (Takara Co., Dalian, China) and performed on an Eppendorf Mastercycler and Mastercycler gradient. The PCR products were assessed by electrophoresis in a 1.5% agarose gel and staining with ethidium bromide. All PCR products were sequenced directly from both directions except for fragment F1. Since fragment F1 encompassed the A+T-rich region and some complex structures (e.g., poly-T and microsatellite-like repeat), giving rise to the failures of sequencing. So we utilized short PCR amplification with the primer pair AF1F and AF1R, and the PCR amplification conditions were as the long PCR amplifications. The PCR product of AF1 was purified with the E.Z.N.A. Gel Extraction Kit (Omega, USA) and ligated into the pMD19-T Vector (Takara Co., Dalian, China). Reconstructive plasmids were isolated from the transformed *E*. *coli* DH5α competent cells and sequenced with the primers M13-F and M13-R. All fragments were sequenced using ABI BigDye ver. 3.1 dye terminator sequencing technology and run on ABI PRISM 3730×1 capillary sequencers.

### Sequence analysis and gene annotation

The whole mitogenome of *S*. *recurvalis* was assembled and completed by aligning the overlapping sequences of neighboring fragments using CLUSTAL X [33]. The 13 PCGs, two rRNA genes, and the A+T-rich region were identified by comparison with the homologous lepidopteran mitogenomes sequences (e.g., *Cnaphalocrocis medinalis*, NC_015985 and *Maruca vitrata*, NC_024099). The nucleotide sequences of 13 PCGs were translated into amino acid sequences on the basis of the invertebrate mitochondrial genetic code. The A+T content of nucleotide sequences and relative synonymous codon usage (RSCU) were calculated using the MEGA ver. 6.0 [34]. The AT skewness was calculated according to the formula: AT skew = [A-T] / [A+T] [35]. The secondary structure of *rrnL* and *rrnS* were drawn by XRNA 1.2.0 b (developed by B. Weiser and available at http://rna.ucsc.edu/rnacenter/xrna/xrna.html). The tRNA genes and secondary structures were identified using the tRNAscan-SE ver. 1.21 (http://selab.janelia.org/tRNAscan-SE/) [36]. The secondary structures of two tRNA^Ser^ genes, which we were unable to predict by using the tRNAscan-SE, were analyzed by comparison with the nucleotide sequences of the tRNA genes in the Crambidae (e.g., *Cnaphalocrocis medinalis*, NC_015985 and *Dichocrocis punctiferalis*, JX448619). The tRNA genes secondary structures were drawn using DNA-SIS ver. 2.5 (Hitachi Engineering, Tokyo, Japan).

### Phylogenetic analysis

Phylogenetic analysis was performed based on the 13 PCGs of the complete mitogenome of *S*. *recurvalis* and 54 other lepidopteran mitogenomes downloaded from GenBank (Table 2). The mitogenomes of *Anopheles gambiae* (NC_002084) [37] and *Drosophila melanogaster* (NC_001709) [38] were used as outgroups. Nucleotide sequences of the 13 PCGs from the mitogenomes of the 54 lepidopteran, two outgroup species, and *S*. *recurvalis* were translated into amino acid sequences. They were aligned with CLUSTAL X using default settings, and then back-translated into nucleotide alignments, then the unaligned and unmatched regions were removed, and the remaining nucleotide alignments were concatenated together. Nucleotide sequences of two rRNA genes from the mitogenomes of the 57 species were aligned with CLUSTAL X using default settings, the unaligned and unmatched regions were removed, and then the concatenated nucleotide sequences were combined to the end of the aligned nucleotide of 13 PCGs respectively. The concatenated nucleotide alignments of 13 PCGs and 13 PCGs+2 rRNAs yielded a nucleotide matrix of 10,719 bp and 12072 bp in length, respectively, which were used for phylogenetic analysis with BI and ML methods.

**Table 2:** Comparisons characteristics of the Lepidoptera and two outgroup species mitogenomes.

Superfamily	Species	Whole mitogenome			PCGs		lrRNA		srRNA		A+T-rich region		Acc.number
		Size (bp)	A+T (%)	A+T skew	Codon no.	A+T (%)	Size (bp)	A+T (%)	Size (bp)	A+T (%)	Size (bp)	A+T (%)	
Bombycoidea	*Actias selene*	15236	78.9	-0.023	3725	77.3	1364	83.6	762	84.0	339	87.9	NC_018133
	*Antheraea pernyi*	15566	80.2	-0.021	3729	78.5	1369	83.8	775	81.1	552	90.4	NC_004622
	*Bombyx mandarina*	15928	81.7	0.055	3718	79.6	1377	84.8	783	86.0	747	95.2	NC_003395
	*Bombyx mori*	15656	81.4	0.059	3716	79.5	1378	83.4	783	85.6	494	95.5	AB070264
	*Eriogyna pyretorum*	15327	80.8	-0.031	3727	79.3	1338	84.6	778	84.4	358	92.2	NC_012727
	*Samia cynthia ricini*	15384	79.8	-0.006	3728	78.2	1358	84.0	779	83.8	361	90.9	NC_017869
	*Sphinx morio*	15299	81.2	0.001	3716	79.8	1379	84.6	773	85.2	316	92.7	NC_020780
Geometroidea	*Biston panterinaria*	15516	79.6	0.077	3726	77.3	1474	85.6	787	84.8	349	93.1	NC_020004
	*Phthonandria atrilineata*	15499	81.5	0.007	3722	79.0	1400	85.7	803	86.3	457	98.3	NC_010522
Noctuoidea	*Gynaephora menyuanensis*	15770	81.5	0.003	3728	79.7	1420	84.2	891	85.5	449	93.3	NC_020342
	*Helicoverpa armigera*	15347	81.0	0.001	3721	79.4	1395	84.7	794	85.9	328	95.1	NC_014668
	*Hyphantria cunea*	15481	80.4	0.009	3721	77.5	1426	85.0	808	85.5	357	95.0	NC_014058
	*Lymantria dispar*	15569	79.9	0.016	3732	77.8	1351	84.2	799	85.2	435	96.1	NC_012893
	*Ctenoplusia agnata*	15261	81.1	0.012	3732	79.8	1328	84.3	784	85.5	334	93.4	KC414791
Papilionoidea	*Apatura metis*	15236	80.4	-0.012	3702	78.9	1333	84.5	779	84.8	394	92.1	NC_015537
	*Aporia crataegi*	15140	81.2	-0.024	3707	79.9	1326	85.4	779	85.5	354	95.2	NC_018346
	*Argynnis hyperbius*	15156	80.8	-0.025	3715	79.4	1330	84.4	778	85.2	349	95.4	NC_015988
	*Artogeia melete*	15140	79.8	0.012	3711	81.4	1319	83.5	777	85.5	351	89.2	EU597124
	*Athyma sulpitia*	15268	82.0	-0.047	3730	80.6	1319	84.7	779	85.8	349	94.6	JQ347260
	*Acraea issoria*	15245	79.8	-0.023	3713	78.0	1331	83.8	788	83.8	430	96.0	NC_013604
	*Calinaga davidis*	15267	80.4	-0.044	3734	78.9	1337	83.8	773	85.4	389	92.0	NC_015480
	*Hebomoia glaucippe*	15701	79.9	-0.036	3718	78.0	1339	84.6	777	85.2	899	92.2	NC_021123
	*Heliconius melpomene melpomene*	15325	81.7	-0.037	3695	80.2	1364	85.6	779	85.1	268	95.9	HE579083
	*Kallima inachus*	15183	80.3	-0.013	3719	79.2	1335	82.8	774	85.1	376	92.0	NC_016196
	*Melanitis leda*	15122	79.8	-0.037	3719	82.4	1332	84.0	774	85.0	314	89.5	JF905446
	*Papilio maackii*	15357	80.7	-0.014	3716	79.2	1326	83.7	774	85.4	514	92.8	KC433408
	*Papilio machaon*	15185	80.3	-0.031	3716	79.0	1319	83.6	773	84.2	362	92.5	NC_018047
	*Pieris rapae*	15157	79.7	0.013	3706	78.2	1320	84.0	764	85.0	393	91.6	NC_015895
	*Protantigius superans*	15248	81.7	-0.036	3709	80.3	1331	85.1	739	85.5	361	93.6	NC_016016
	*Spindasis takanonis*	15349	82.4	0.004	3714	81.0	1333	85.6	777	84.7	371	94.6	NC_016018
	*Coreana raphaelis*	15314	82.7	-0.047	3707	81.5	1330	85.3	777	85.9	375	94.1	NC_007976
Pyraloidea	*Cnaphalocrocis medinalis*	15388	81.9	-0.015	3724	80.5	1389	84.9	781	86.2	339	95.9	NC_015985
	*Chilo suppressalis*	15395	80.7	0.008	3729	78.8	1383	84.2	788	86.2	348	95.4	NC_015612
	*Corcyra cephalonica*	15273	80.4	-0.036	3713	78.9	1355	83.0	778	85.9	351	96.6	NC_016866
	*Diatraea saccharalis*	15490	80.0	0.021	3721	77.8	1412	84.8	781	85.5	335	94.9	NC_013274
	*Dichocrocis punctiferalis*	15355	80.6	-0.025	3716	78.8	1360	84.5	793	86.0	338	96.5	JX448619
	^*※*^ *Ostrinia furnacalis*	14535	80.4	0.032	3714	79.4	1339	85.0	435	82.8	-	-	NC_003368
	^*※*^ *Ostrinia nubilalis*	14535	80.2	0.032	3714	79.1	1339	84.9	434	82.0	-	-	NC_003367
	*Paracymoriza prodigalis*	15326	81.5	0.002	3714	80.0	1389	85.5	781	86.0	343	95.3	NC_020094
	*Scirpophaga incertulas*	15223	77.1	0.031	3718	74.8	1314	82.0	768	84.1	403	92.8	NC_021413
	*Elophila interruptalis*	15351	80.3	-0.015	3712	78.6	1367	84.1	786	85.5	339	93.5	NC_021756
	*Glyphodes quadrimaculalis*	15255	80.8	-0.007	3714	79.2	1350	84.9	779	85.5	327	94.5	NC_022699
	*Ephestia kuehniella*	15294	79.8	-0.048	3714	78.1	1328	84.2	773	84.9	322	93.2	NC_022476
	*Maruca vitrata*	15385	80.7	-0.002	3772	79.3	1304	84.4	765	85.2	341	93.0	NC_024099
	***Spoladea recurvalis***	**15273**	**80.9**	**-0.023**	**3719**	**79.3**	**1384**	**85.3**	**781**	**86.0**	**329**	**93.9**	**KJ739310**
Hesperioidea	*Ctenoptilum vasava*	15468	80.5	-0.029	3706	78.9	1343	84.1	774	85.7	429	88.1	NC_016704
	*Erynnis montanus*	15530	81.8	-0.001	3717	80.0	1408	85.1	780	85.8	389	95.1	KC659955
Tortricoidea	*Adoxophyes honmai*	15679	80.4	-0.001	3733	78.4	1387	83.6	779	85.4	489	94.3	NC_008141
	*Adoxophyes orana*	15343	80.0	-0.001	3723	78.3	1430	84.8	804	85.3	331	92.8	JX872403
	*Cydia pomonella*	15253	80.1	-0.004	3723	78.5	1361	83.8	813	85.5	327	96.0	NC_020003
	*Grapholita molesta*	15716	80.9	-0.004	3736	78.8	1377	84.8	772	85.4	770	96.0	NC_014806
	*Spilonota lechriaspis*	15368	81.2	-0.018	3737	80.6	1382	85.2	778	86.3	441	92.7	NC_014294
Hepialoidea	*Ahamus yunnanensis*	15816	82.3	-0.006	3717	80.6	1329	86.0	770	86.1	978	89.4	HM744695
	*Thitarodes renzhiensis*	16173	81.3	-0.001	3717	79.0	1335	85.4	779	85.4	1367	90.5	HM744694
Yponomeutoidea	*Plutella xylostella*	16079	81.4	0.005	3719	79.3	1379	85.0	783	86.1	981	91.6	JF911819
Drosophiloidea	*Drosophila melanogaster*	19517	82.2	0.017	3716	77.2	1325	83.0	786	80.2	4601	95.6	NC_001709
Culicoidea	*Anopheles gambiae*	15363	77.6	0.032	3733	75.9	1325	82.5	800	79.6	519	94.2	NC_002084

※, Incomplete mitogenomes lack the partial *rrnS* gene, the entire A+T-rich region and partial tRNA^Met^ gene. Termination codons were excluded in 13 PCGs.

By using Akaike Information Criterion (AIC) [39], the substitution model selection was calculated using the program Modeltest ver. 3.7 [40]. The TVM+I+G model was chosen as the best-fitting model for BI analysis with the dataset of 13 PCGs, and the second one was GTR+I+G. The GTR+I+G model was chosen as the best-fitting model for BI analysis with the dataset of 13 PCGs+2 rRNAs. The BI analysis was performed using MrBayes ver. 3.1 [41] under the following conditions: 10,000,000 generations, four independent chains (one cold chain and three hot chains) with tree sampling every 100 generations and a burn-in of 2500 trees. The confidence values of the BI tree were expressed as the Bayesian posterior probabilities. The posterior probabilities more than 0.9 were considered strongly-supported [42].

The ML analysis was performed using the PHYML online web server (http://www.atgc-montpellier.fr/phyml/) [43]. The setting conditions in the substitution model block were as follows: (a) Substitution model–“GTR”, (b) Equilibrium frequencies–“empirical”, (c) Transition/ transversion ratio–“fixed”, Proportion of invariable sites–“estimated”, Number of substitution rate categories–“4”, and (e) Gamma shape parameter–“estimated”. In the tree searching block, the setting conditions were (f) Starting tree(s)–“BIONJ”, (g) Type of tree improvement–“SPR”, (h) Number of random starting tree–“no”, (i) Optimise topology–“yes”, and (j) Optimise branch lengths–“yes”. Finally, in the branch support block, the settings conditions were (k) Fast likelihood-based method–“no”, (l) Perform bootstrap–“yes”, and (m) Perform bootstrap–“1000”. In ML analysis, nodes with bootstrap proportions over 70% were interpreted as well-supported [44]. The phylogenetic trees were viewed and edited using Figtree ver. 1.4 [45].

## Results and Discussion

### Genome structure and organization

**Fig 1:**
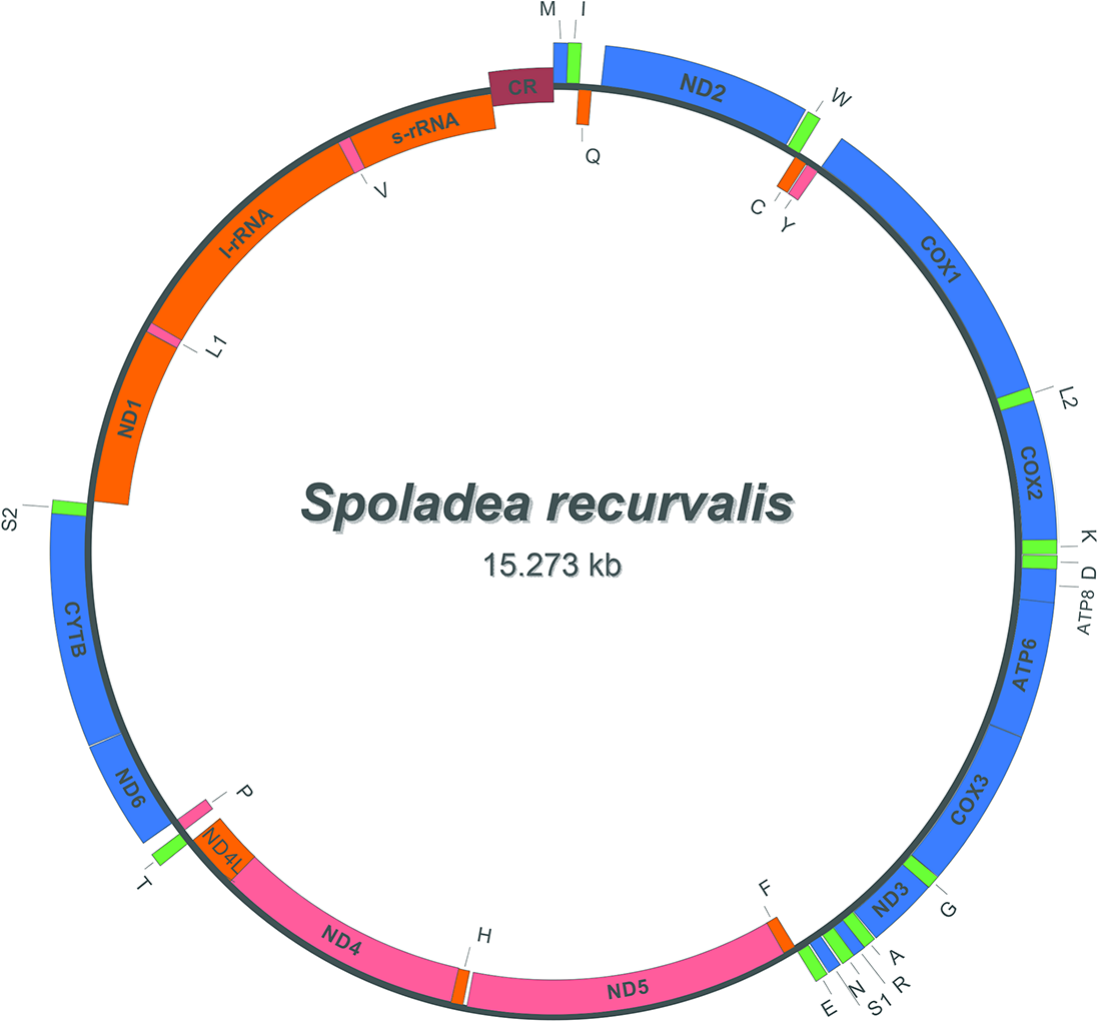
Map of the mitochondrial genome of *Spoladea recurvalis*. Genes coded on the J strand (clockwise orientation) are blue- or green-colored, while the genes coded on the N strand (anti-clockwise orientation) are pink- or orange-colored. COX1, COX2 and COX3 refer to the cytochrome c oxidase subunits; CYTB refers to cytochrome B; ATP6 and ATP8 refer to ATP synthase subunits 6 and 8 genes; and ND1–ND6 and ND4L refer to the NADH dehydrogenase subunit 1–6 and 4L genes. tRNA genes are denoted as one-letter symbols according to the IUPAC-IUB single-letter amino acid codes: L1, L2, S1 and S2 refer to tRNA^Leu(CUN)^, tRNA^Leu(UUR)^, tRNA^Ser(AGY)^, and tRNA^Ser(UCN)^, respectively. CR refering to the A+T rich region and is brown-colored.

The mitogenome of *S*. *recurvalis* was found to be a circular molecule with 15,273 bp in length, which is well within the range of other lepidopteran mitogenomes, with lengths ranging from 15,122 bp in *M*. *leda* to 16,173 bp in *T*. *renzhiensis* (Table 2). The mitogenome of *S*. *recurvalis* contained the typical set of 37 typical mitochondrial genes (13 PCGs, 22 tRNA genes, and two rRNA genes), and a major non-coding region known as the A+T-rich region, as has been found in other lepidopteran mitogenomes. Twenty-three genes were coded on the majority strand (J-strand) and the rest were coded on the minority strand (N-strand) (Table 3 and Fig 1). This mitogenome was submitted to GenBank under the accession number KJ739310.

**Table 3:** Annotation of the *Spoladea recurvalis* mitogenome.

Gene	strand	Location	Size	Inc	Anticodon	Start codon	Stop codon
tRNA^Met (M)^	J	1–69	69		CAT		
tRNA^Ile (I)^	J	70–134	65	-3	GAT		
tRNA^Gln (Q)^	N	132–200	69	48	TTG		
ND2	J	249–1262	1014	13		ATT	TAA
tRNA^Trp (W)^	J	1276–1343	68	-8	TCA		
tRNA^Cys (C)^	N	1336–1404	69	7	GCA		
tRNA^Tyr (Y)^	N	1412–1477	66	3	GTA		
COX1	J	1481–3011	1531			CGA	T-
tRNA^Leu(UUR)^	J	3012–3078	67		TAA		
COX2	J	3079–3763	685	-3		ATG	T-
tRNA^Lys (K)^	J	3761–3831	71	6	CTT		
tRNA^Asp (D)^	J	3838–3904	67		GTC		
ATP8	J	3905–4066	162	-7		ATT	TAA
ATP6	J	4060–4734	675	3		ATG	TAA
COX3	J	4738–5526	789	2		ATG	TAA
tRNA^Gly (G)^	J	5529–5593	65		TCC		
ND3	J	5594–5947	354	6		ATT	TAA
tRNA^Ala (A)^	J	5954–6018	65	1	TGC		
tRNA^Arg (R)^	J	6018–6081	64		TCG		
tRNA^Asn (N)^	J	6082–6150	69	11	GTT		
tRNA^Ser(AGY)^	J	6162–6229	68	10	GCT		
tRNA^Glu (E)^	J	6240–6305	66	-2	TTC		
tRNA^Phe (F)^	N	6304–6371	68		GAA		
ND5	N	6372–8094	1723	21		ATT	T-
tRNA^His (H)^	N	8116–8181	66		GTG		
ND4	N	8182–9521	1337	-1		ATG	TA-
ND4L	N	9521–9814	294	2		ATG	TAA
tRNA^Thr (T)^	J	9817–9883	67		TGT		
tRNA^Pro (P)^	N	9884–9949	66	2	TGG		
ND6	J	9952–10485	534	6		ATC	TAA
CYTB	J	10492–11640	1149	-2		ATG	TAA
tRNA^Ser(UCN)^	J	11639–11704	66	15	TGA		
ND1	N	11720–12658	939	1		ATG	TAA
tRNA^Leu(CUN)^	N	12660–12728	69	-16	TAG		
*rrnL*	N	12713–14096	1384				
tRNA^Val (V)^	N	14097–14163	67		TAC		
*rrnS*	N	14164–14944	781				
A+T-rich Region		14945–15273	329				

Inc, intergenic nucleotides, negative values refer to overlapping nucleotides.

The gene order and orientation of the *S*. *recurvalis* mitogenome were identical to that of other reported ditrysian lepidopteran mitogenomes, but differed from non-ditrysian groups with the ancestral arrangement of tRNA^Ile^–tRNA^Gln^–tRNA^Met^, such as the species *Thitarodes renzhiensis* and *Ahamus yunnanensis* in Hepialoidea [46]. All the ditrysian lineages of lepidopteran mitogenomes are characterized by the gene order tRNA^Met^–tRNA^Ile^–tRNA^Gln^, revealing a translocation of tRNA^Met^ to a position 5'-upstream of tRNA^Ile^, which differs from the hypothesized ancestral gene order of insects [47]. This suggests that the mitogenome arrangement of the lepidopteran insects may have evolved independently after splitting from a stem lineage of insects [48].

The nucleotide composition (A 39.5%, G 7.8%, T 41.4% and C 11.3%) of the *S*. *recurvalis* mitogenome indicated a high A+T content of 80.9%, which is well within the range of lepidopteran mitogenomes, which vary from 77.0% in *S*. *incertulas* to 82.7% in *C*. *raphaelis*, similar to that of *C*. *medinalis* (80.9%). The mitogenome A+T content was 79.3% in 13 PCGs, 85.3% in *rrnL* genes, 86.0% in *rrnS* genes, and 93.9% in the A+T-rich region, respectively. These values were consistent with the high values found in other lepidopteran mitogenomes (Table 2). The AT skewness of the mitogenome was -0.023, indicating the occurrence of more T nucleotides than A nucleotides, as has been found in other lepidopteran mitogenomes, with the values ranging from -0.048 in *E*. *kuehniella* to 0.059 in *B*. *mori* (Table 2).

### Protein-coding genes

The PCGs regions of the *S*. *recurvalis* mitogenomes were consistent with those of other lepidopteran mitogenomes. Nine of the 13 PCGs (ND2, COX1, COX2, ATP8, ATP6, COX3, ND3, ND6, and CYTB) were coded on the majority strand (J-strand), and the remaining four PCGs (ND5, ND4, ND4L, and ND1) were coded on the minority strand (N-strand) (Table 3 and Fig 1). All PCGs initiated with a canonical start codon ATN with the exception of COX1, which may use an arginine CGA as the start codon. Specifically, seven PCGs (COX2, ATP6, COX3, ND4, ND4L, CYTB, and ND1) started with ATG, four PCGs (ND2, ATP8, ND3 and ND5) started with ATT, and one PCG (ND6) started with ATC. As for stop codon, nine PCGs (ND2, ATP8, ATP6, COX3, ND3, ND4L, ND6, CYTB and ND1) terminated with the standard stop codon TAA, whereas COX1, COX2 and ND5 used single T nucleotide, and ND4 used TA nucleotides as an incomplete stop codon. The non-canonical termination codons will be corrected by post-transcriptional modifications, such as polyadenylation, which occur during the mRNA maturation process [49,50]. The partial stop codons observed in most lepidopteran species minimize the intergenic spacers and gene overlaps may be one strategy for the selection of a stop codon [51].

The start codons for the COX1 gene of the lepidopteran insects have been the source of controversy in current studies. In other insects groups, some canonical codons were proposed as the COX1 start codon, such as TTA [52], TCG [53], TTG [54], and ACG [55]. In addition, some tetranucleotides, such as TTAG [48,56], ATAA [57–60], and some hexanucleotides, such as ATTTAA [37,61,62], TATCTA [63], and TATTAG [20,64,65], located immediately upstream of the CGA, have also been proposed as the start codons for the COX1 gene. However, a recent study, based on the transcript information of *Anopheles funestus* (Diptera), revealed that the translation initiation codon for the COX1 gene was TCG (Serine), rather than the atypical and longer codons which have been proposed for several other insects [66]. Data from the transcript map, with expressed sequence tags (ESTs) for the mitochondrial genome annotation of the legume pod borer *Maruca vitrata* (Lepidoptera: Crambidae), showed that the COX1 gene started with the CGA codon for arginine [67]. This start codon has been found previously well conserved in other lepidopteran species [68]; therefore, we tentatively designated CGA as the start codon of COX1 gene.

### Codon usage

The relative synonymous codon usage (RSCU) value of the *S*. *recurvalis* mitogenome is summarized in Table 4. Excluding all initiation and termination codons, the 13 PCGs were 11,118 bp in total length, encoding 3,706 amino acid residues. The codons CCG, UGG, CGC, CGG, AGC, GGC, and AGG were not presented in these PCGs. The codons UUA (12.6%), AUU (12.0%), UUU (9.2%), AUA (7.0%), and AAU (6.7%) were the five most frequently used codons in the *S*. *recurvalis* mitogenome, accounting for 47.5%. These codons were all composed of A or U nucleotide, indicating the high biased usage of A and T nucleotides in the *S*. *recurvalis* PCGs. Likewise, the most frequent amino acids in the *S*. *recurvalis* mitochondrial proteins were Leu2 (13.0%), Ile (12.6%), Phe (10.1%), Met (7.0%), and Asn (6.6%), accounting for 49.3%. The least amino acid was Cys (0.8%). Codon usage of PCGs showed a significant bias of high A + T content, which played a major role in the A+T bias of the entire mitogenome.

**Table 4:** Codon usage in the PCGs of *Spoladea recurvalis* mitogenome.

Codon	Count	%	RSCU	Codon	Count	%	RSCU	Codon	Count	%	RSCU	Codon	Count	%	RSCU
UUU(F)	341	9.2	1.82	UCU(S)	109	2.9	2.63	UAU(Y)	181	4.9	1.88	UGU(C)	26	0.7	1.73
UUC(F)	33	0.9	0.18	UCC(S)	11	0.3	0.27	UAC(Y)	12	0.3	0.12	UGC(C)	4	0.1	0.27
UUA(L)	466	12.6	5.29	UCA(S)	87	2.3	2.10	UAA(*)	-	-	-	UGA(W)	95	2.6	2.00
UUG(L)	13	0.4	0.15	UCG(S)	5	0.1	0.12	UAG(*)	-	-	-	UGG(W)	0	0.0	0.00
CUU(L)	29	0.8	0.33	CCU(P)	66	1.8	2.10	CAU(H)	59	1.6	1.74	CGU(R)	17	0.5	1.31
CUC(L)	4	0.1	0.05	CCC(P)	14	0.4	0.44	CAC(H)	9	0.2	0.26	CGC(R)	0	0.0	0.00
CUA(L)	14	0.4	0.16	CCA(P)	46	1.2	1.46	CAA(Q)	58	1.6	1.87	CGA(R)	35	0.9	2.69
CUG(L)	3	0.1	0.03	CCG(P)	0	0.0	0.00	CAG(Q)	4	0.1	0.13	CGG(R)	0	0.0	0.00
AUU(I)	445	12.0	1.91	ACU(T)	81	2.2	2.13	AAU(N)	231	6.2	1.88	AGU(S)	33	0.9	0.80
AUC(I)	21	0.6	0.09	ACC(T)	5	0.1	0.13	AAC(N)	15	0.4	0.12	AGC(S)	0	0.0	0.00
AUA(M)	258	6.7	1.82	ACA(T)	65	1.8	1.71	AAA(K)	100	2.7	1.90	AGA(S)	87	2.3	2.10
AUG(M)	12	0.3	0.18	ACG(T)	1	0.0	0.03	AAG(K)	5	0.1	0.10	AGG(S)	0	0.0	0.00
GUU(V)	78	2.1	2.28	GCU(A)	81	2.2	2.45	GAU(D)	56	1.5	1.81	GGU(G)	50	1.3	1.02
GUC(V)	3	0.1	0.09	GCC(A)	4	0.1	0.12	GAC(D)	6	0.2	0.19	GGC(G)	0	0.0	0.00
GUA(V)	54	1.5	1.58	GCA(A)	43	1.2	1.30	GAA(E)	72	1.9	1.85	GGA(G)	127	3.4	2.58
GUG(V)	2	0.1	0.06	GCG(A)	4	0.1	0.12	GAG(E)	6	0.2	0.15	GGG(G)	20	0.5	0.41

A total of 3706 codons were analyzed excluding all initiation termination codons.

RSCU, relative sunonymous codon usage.

### Transfer and ribosomal RNA genes

**Fig 2:**
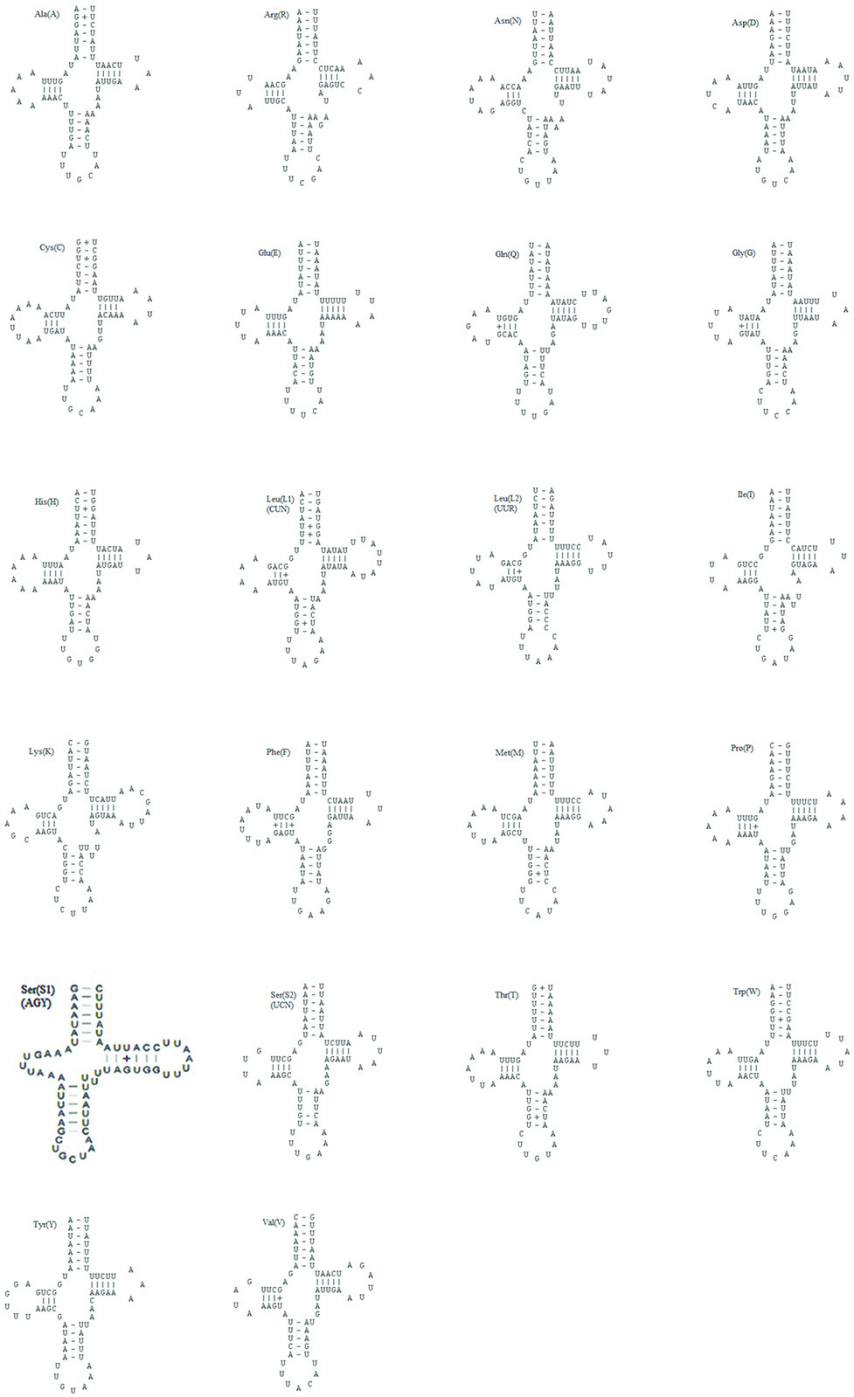
Putative secondary structures for the 22 tRNA genes of *Spoladea recurvalis* mitogenome.

The mitogenome of *S*. *recurvalis* contained the typical set of 22 tRNAs genes as have been found in most lepidopteran mitogenomes. The tRNAs genes were scattered throughout the circular molecule and range from 64 bp (tRNA^Arg^) to 71 bp (tRNA^Lys^) in size (Table 3). Fourteen tRNA genes were coded on the J-strand and the other eight on the N-strand, as with other lepidopteran mitogenomes (Table 3 and Fig 1). The putative secondary structure of the *S*. *recurvalis* tRNAs are shown in Fig 2. All tRNA genes were folded into the typical cloverleaf secondary structures, except for the tRNA^Ser (AGY)^ gene, in which the dihydrouridine (DHU) arm was simplified as a loop, which has been observed in several other metazoan species, including insects [2]. The anticodons of the tRNA genes were identical to those most reported in insect mitogenomes.

A total of 26 pairs of mismatched base pairs were found in 16 tRNA genes, including ten pairs in the amino acid acceptor stems, eight pairs in the DHU stems, seven pairs in the anticodon stems, and one pair in the pseudouridine (TΨC) stems. The 21 U–G mismatched bases may have formed weak bonds, while the other five U–U mismatches have not. The mismatched base pairs in tRNAs are modified via RNA-editing mechanisms that are well known in arthropod mitogenomes [69].

As seen in other insect mitogenomes, two rRNA genes (*rrnL* and *rrnS*) were present in the *S*. *recurvalis* mitogenome. The *rrnL* gene was located between the tRNA^Leu(CUN)^ and tRNA^Val^, and the *rrnS* gene was located between the tRNA^Val^ and the A + T-rich region. The lengths of the *rrnL* gene and *rrnS* gene were 1384 bp and 781 bp, respectively, which are well within the lengths reported for these genes for other lepidopteran mitogenomes. The A + T contents of the *rrnL* gene and *rrnS* gene of *S*. *recurvalis* mitogenome were 85.3% and 86.0%, respectively. These values are also well within the range of other lepidopteran mitogenomes. The lengths of the *rrnL* genes varied from 1304 bp to 1474 bp and the A+T contents varied from 82.0% to 85.6%. The lengths of the *rrnS* genes varied from 739 bp to 891 bp and the A+T contents varied from 81.1% to 86.3% (Table 2).

**Fig 3:**
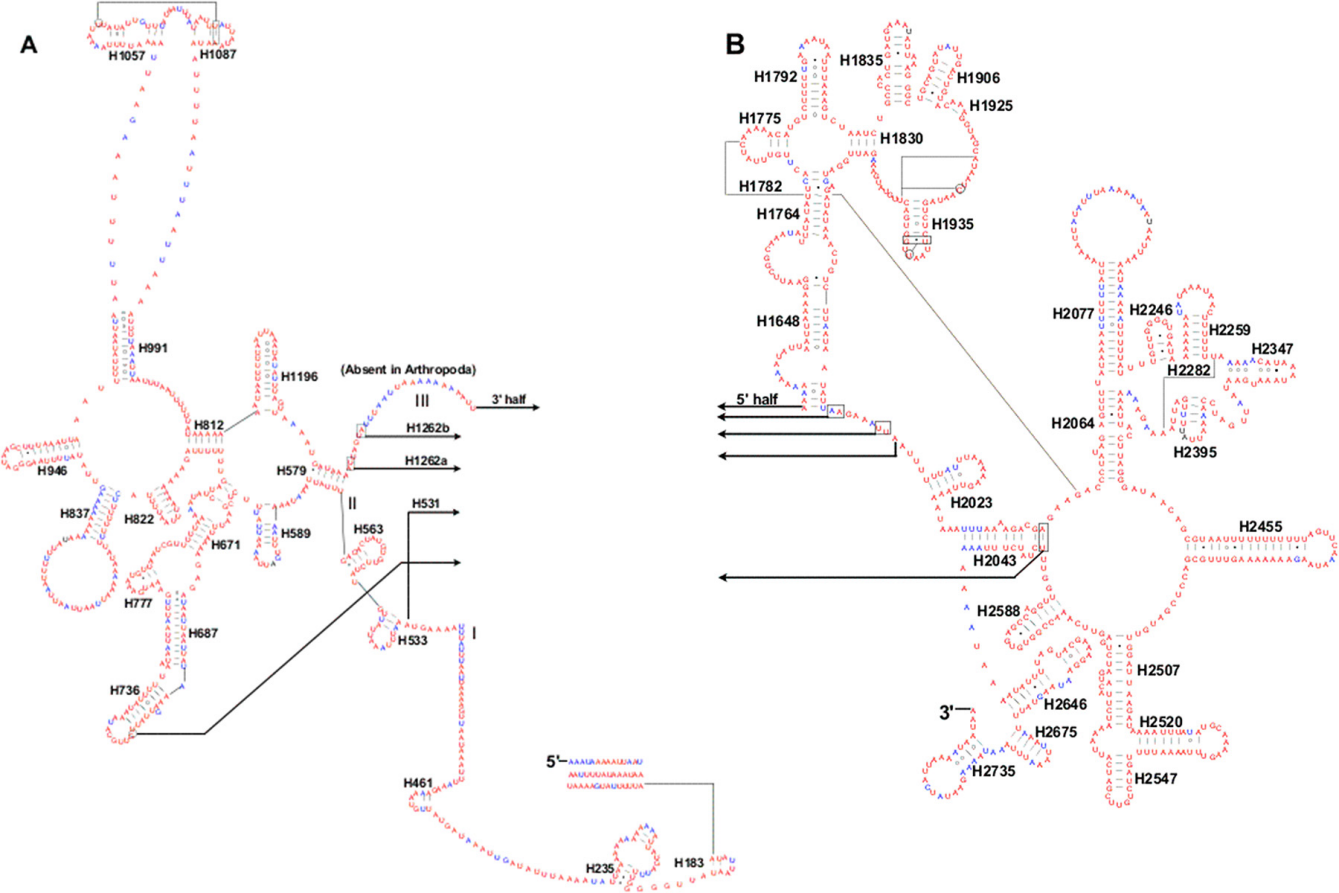
Predicted *rrnL* secondary structure in the *S*. *recurvalis* mitogenome. Tertiary interactions and base triples are connected by continuous lines. Base-pairing is indicated as follows: Watson-Crick pairs by lines, wobble GU pairs by dots and other non-canonical pairs by circles. A represents the 5’ half of *rrnL*; B represents the 3’ half of *rrnL*.

Both the secondary structure of *rrnL* and *rrnS* broadly conformed to the secondary structure models proposed for these genes from other insects [7,17,70–72]. In the mitogenome of *S*. *recurvalis*, six domains with 49 helices were present in the *rrnL* secondary structure (Fig 3A and 3B), as in *M*. *sexta* [7], *C*. *medinalis* [17], *C*. *suppressalis* [17], *A*. *emma* [70], *L*. *malifoliella* [71] and *P*. *xylostella* [72]. An internal 31bp large loop was located among the H991, H1057, and H1087, which is similar to that of *C*. *medinalis*, *C*. *suppressalis*, *A*. *emma*, *L*. *malifoliella* and *P*. *xylostella* but different from that of *M*. *sexta*. As in the *rrnL* secondary structure of *M*. *sexta*, *C*. *medinalis*, *C*. *suppressalis*, *A*. *emma*, and *L*. *malifoliella*, but differs from that of *P*. *xylostella* which contains a (TA)_8_ microsatellite-like repeat inserted into the loop region of H2347. This region is highly variable within Lepidoptera and a consistent secondary structure for it has not been found within the available lepidopteran mitogenomes [7].

**Fig 4:**
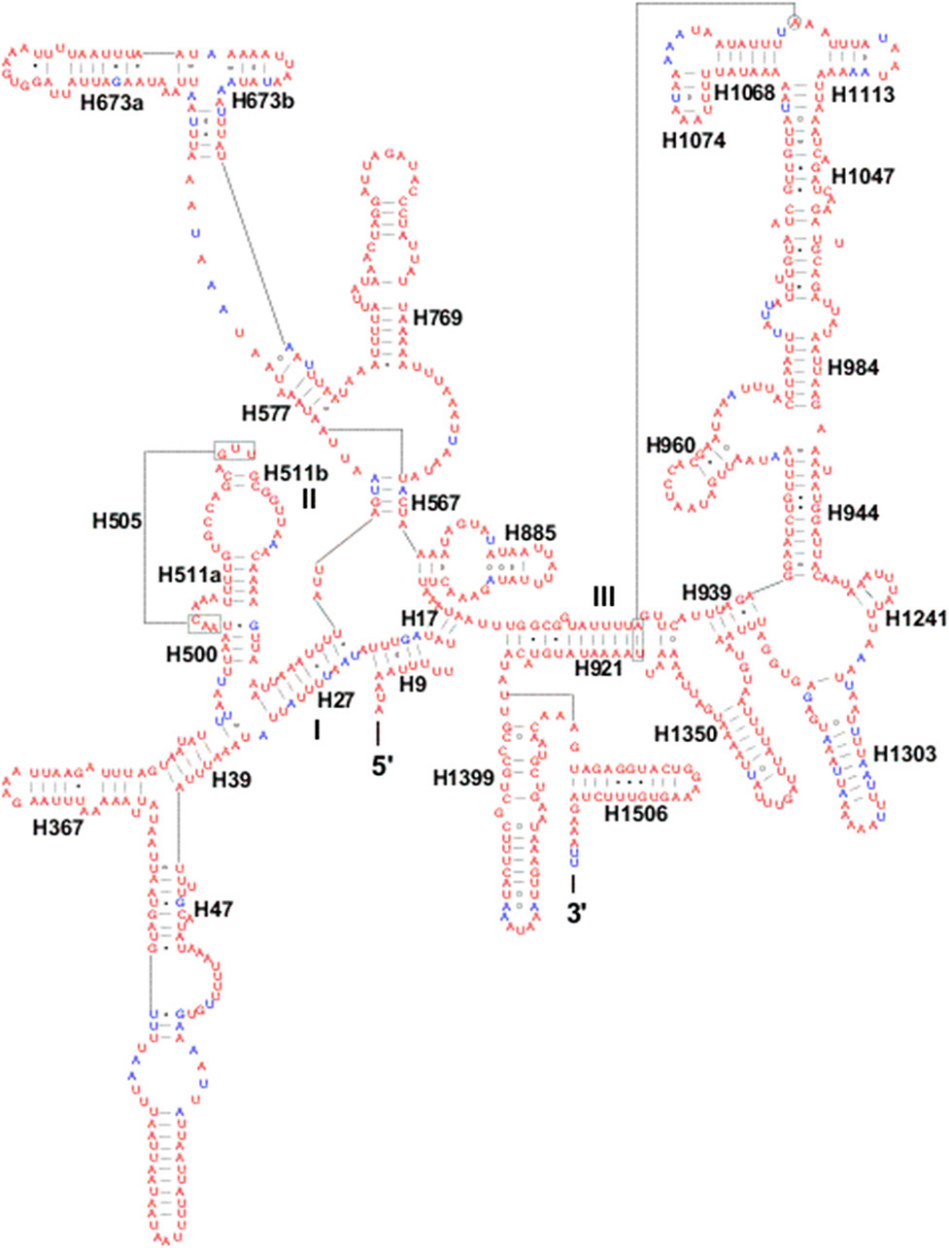
Predicted *rrnS* secondary structure in the *S*. *recurvalis* mitogenome. Tertiary interactions and base triples are connected by continuous lines. Base-pairing is indicated as follows: Watson-Crick pairs by lines, wobble GU pairs by dots and other non-canonical pairs by circles.

Three domains with 29 helices were present in the *rrnS* secondary structure of *S*. *recurvalis* mitogenome (Fig 4). A small loop was located in the H47 region of the *rrnS* gene compared to that of *M*. *sexta* and *P*. *xylostella*. This region has been found to be variable within lepidopteran species [7], which has been used to predict the phylogenetic relationships when it is combined with H39 and H367 [73].

The H673, H1047, H1068, and H1074 present in the *rrnS* secondary structure of the *S*. *recurvalis* mitogenome were similar in length and secondary structures to those of *C*. *medinalis* and *C*. *suppressalis*, but different from those of *M*. *sexta*, indicating they are also variable regions in the secondary structure of *rrnS* gene within lepidopteran species [7]. The secondary structures of two rRNAs were predicted mainly based on sequence comparison and mathematical methods. The region in *rrnS* contains the H1047, H1068, and H1074 may yield several possible secondary structures, but it is not ascertained which one may be utilized among these structures [7].

### Non-coding and overlapping region

The non-coding region of the *S*. *recurvalis* mitogenome was 157 bp in total, which consisted of 17 non-coding regions, ranging from 1 to 48 bp and including 5 major non-coding regions of more than 10 bp (Table 3). The longest intergenic spacer (Spacer1, 48bp) was located between the tRNA^Gln^ and ND2, with an extremely high richness in A + T nucleotides (95.8%). This spacer has been a feature common reported in the other lepidopteran mitogenomes, which has been sequenced to date, but which has not been found in non-lepidopteran insect species [7]. Spacer 2 (13 bp) was located between the ND2 and tRNA^Trp^, which was only smaller than the 18 bp space of *Hyphantria cunea* in the similar location in lepidopteran mitogenomes [74]. Spacer 3 (11 bp) was located between the tRNA^Asn^ and tRNA^Ser (AGY)^, and Spacer 4 (21 bp) was located between the ND5 and tRNA^His^. Spacer 5 (15 bp), which was located between the tRNA^Ser(UCN)^ and ND1, contained the motif ‘‘ATACTAA”, which represents a conserved feature across lepidopteran insects [7]. The motif has been proposed as a possible mitochondrial transcription termination peptide-binding site (mtTERM protein) [4].

Eight overlapping sequences of the *S*. *recurvalis* mitogenome were found in eight different locations, ranging from 1 to 16 bp with a total of 42 bp. The longest overlapping sequence (16 bp) was located between the tRNA^Leu(CUN)^ and *rrnL*, and the second longest overlapping sequence (8 bp) was located between the tRNA^Trp^ and tRNA^Cys^. The third longest overlapping sequence was located between the ATP8 and ATP6 with a seven-nucleotide overlapping sequence (ATGATAA), which has been a common feature reported for many other lepidopteran mitogenomes, and also reported for many animal mtDNAs [1]. A similar-sized overlapping sequence in the same location has been reported in other lepidopteran mitogenomes [70]. The remaining overlapping sequences were all less than 3 bp.

### A + T-rich region

The A + T-rich region of the *S*. *recurvalis* mitogenome was located between the *rrnS* and tRNA^Met^ with a length of 329 bp and with A+T nucleotides accounting for 93.9%, which are within the range of other lepidopteran mitogenomes, which vary from 88.1% in *C*. *vasava* to 98.3% in *P*. *atrilineata* (Table 2). The A + T-rich region has also been found to contain the origin sites for transcription and replication [4].

**Fig 5:**
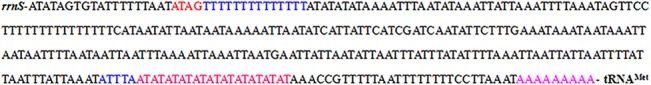
The structure of the A+T-rich region of the *Spoladea recurvalis* mitogenome.

The A+T-rich of the *S*. *recurvalis* mitogenome region was comprised of non-repetitive sequences and had some features in common with other lepidopteran mitogenomes (Fig 5). In *Bombyx*, the O_N_ (the origin of minority or light strand replication) has the motif ATAGA preceded by an 18 bp poly-T stretch and is located 21 bp upstream from the *rrnS* gene [75]. Though the length of the poly-T stretch varies between species, this motif ATAGA is conserved within Lepidoptera [7]. In *S*. *recurvalis*, the motif ATAG was similarly located 19 bp downstream from the *rrnS* gene and followed by a 14 bp poly-T stretch. The poly-T stretch has been postulated to be a transcription control and/or the initiation site of replication [76]. A (AT)_11_ microsatellite-like repeat was preceded by the motif ATTTA located in the 3' end of the A+T-rich region. A poly-A element was present upstream of tRNA^Met^ as has been found in most lepidopteran mitogenomes.

### Phylogenetic relationships

**Fig 6:**
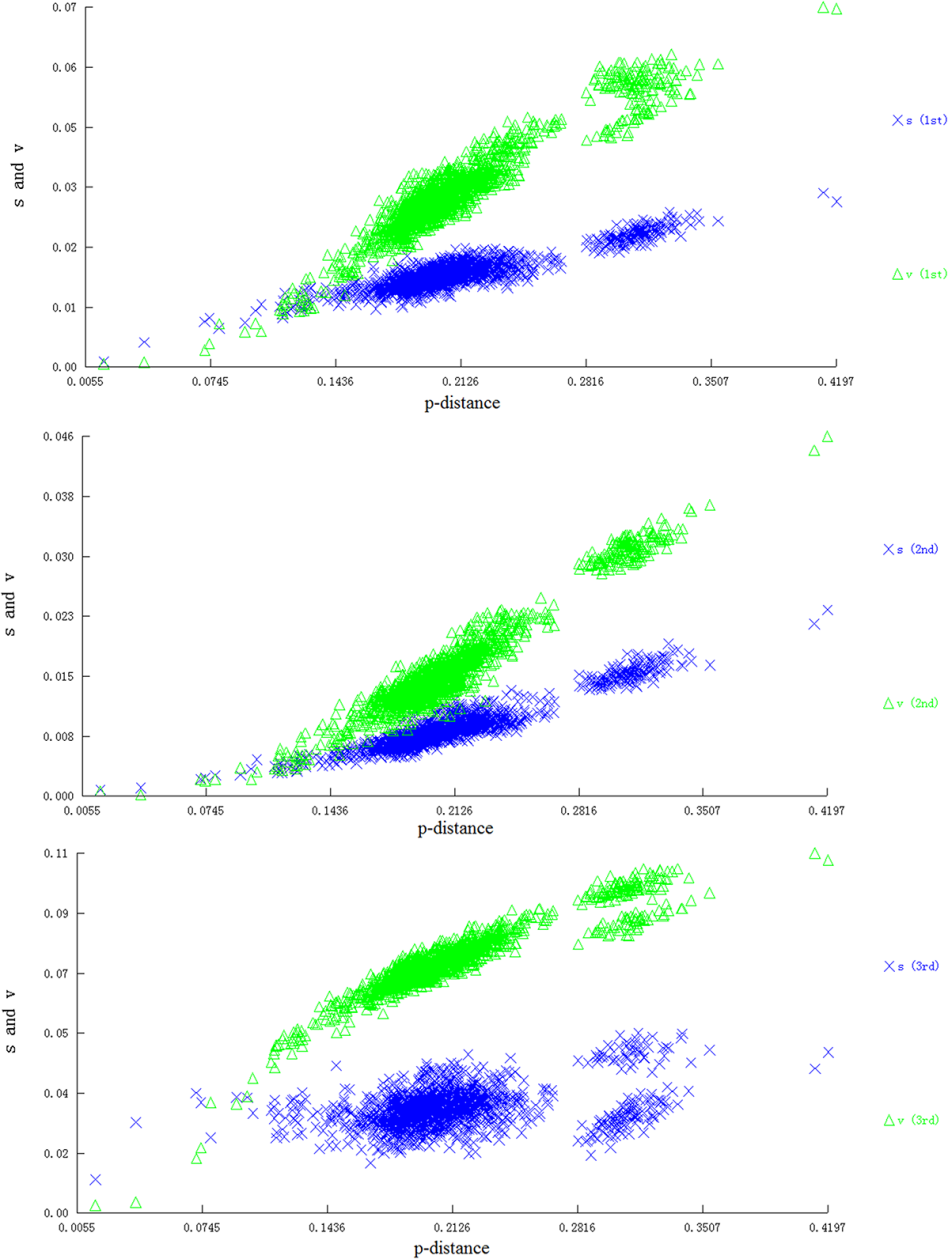
Substitution saturation analysis in the first, second, and third codon positions of 13 PCGs in Lepidoptera (s: transitions; v: transversions).

The estimated Transition/Transversion bias (R) of the first, second, and third codon positions of the 13 PCGs were 0.9, 0.6, and 3.7, respectively. The substitution rates were estimated under the Kimura 2-parameter model in MAGE ver. 6.0 [34]. The transversions and transitions in the first and the second codon position increased linearly with the extension of the phylogeny distance, while the transversions and transitions in the third codon position tended to reach the plateau state (Fig 6).

**Fig 7:**
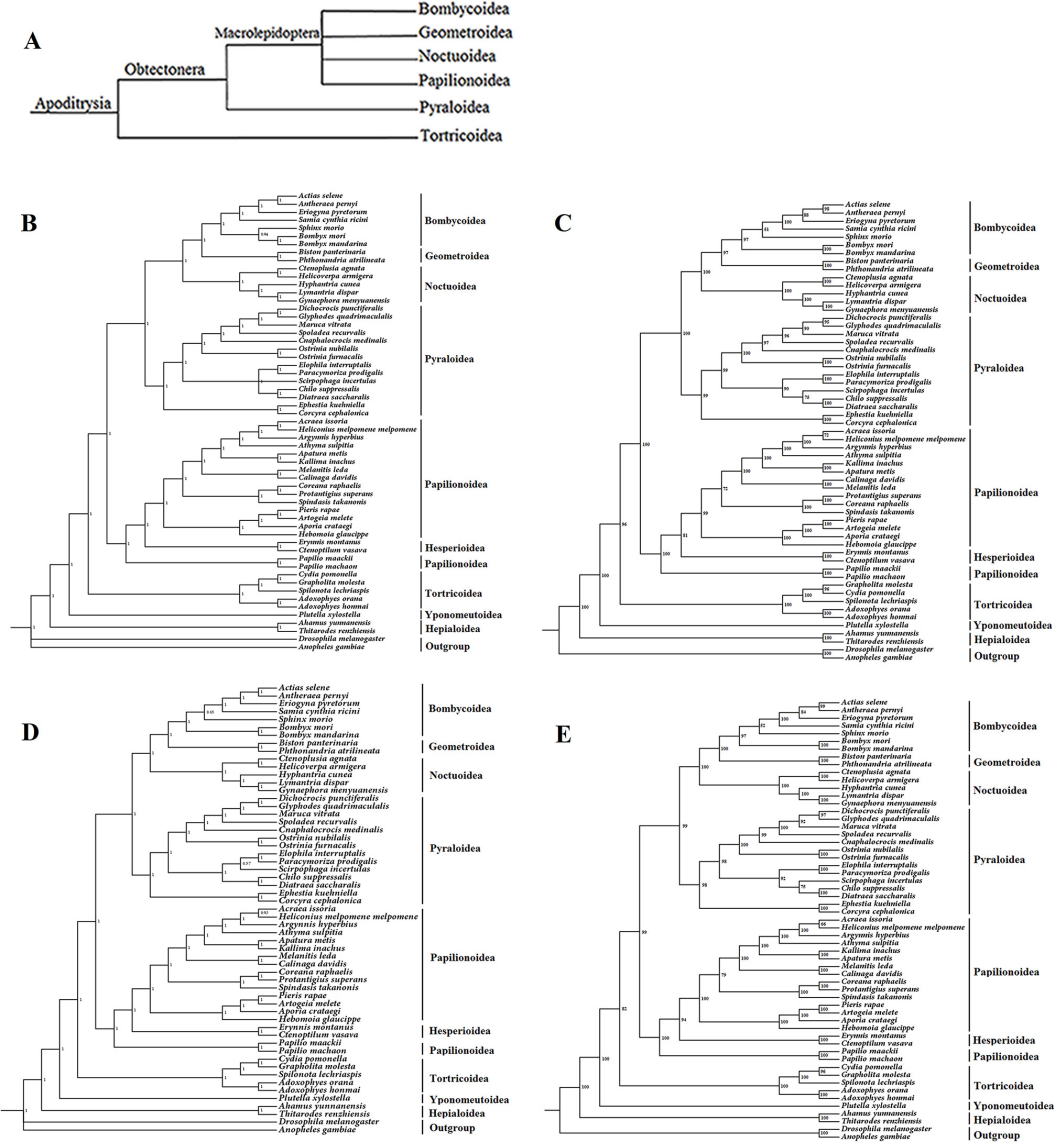
Phylogeny of the Lepidoptera. (A) The recent consensus view of lepidopteran relationships after Kristensen and Skalski (1999). (B) Phylogenetic tree inferred from nucleotide sequences of 13 PCGs using BI method (the numbers abutting branches refer to posterior probabilities). (C) Phylogenetic tree inferred from nucleotide sequences of 13 PCGs using ML method (the numbers abutting branches refer to bootstrap percentages). (D) Phylogenetic tree inferred from nucleotide sequences of 13 PCGs+2 rRNAs using BI method (the numbers abutting branches refer to posterior probabilities). (E) Phylogenetic tree inferred from nucleotide sequences of 13 PCGs+2 rRNAs using ML method (the numbers abutting branches refer to bootstrap percentages). *Drosophila melanogaster* (NC_001709) and *Anopheles gambiae* (NC_002084) were used as outgroups.

In our study, 54 lepidopteran mitogenomes were downloaded from Genebank to reconstruct phylogenetic relationships. The phylogenetic trees were inferred from the concatenated nucleotide sequences of 13 PCGs and 13 PCGs+2 rRNAs using BI and ML methods. The four tree topologies were almost identical to each other and indicated that *S*. *recurvalis* grouped with the other species within the Pyraloidea with strong posterior probabilities and bootstrap support. Only one node was weakly supported in the phylogenetic tree inferred from the dataset of 13 PCGs using ML method, two nodes were weakly supported in the phylogenetic tree inferred from the dataset of 13 PCGs+2 rRNAs using BI method and ML method, respectively (Fig 7B, 7C, 7D and 7E). In our analysis, the subfamilies relationships within the Pyraloidea are consistent with previous studies based on molecular and morphological characteristics [42,77,78].

The 54 species represent nine Lepidoptera superfamilies: Tortricoidea, Bombycoidea, Noctuoidea, Pyraloidea, Geometroidea, Hesperioidea, Papilionoidea, Yponomeutoidea and Hepialoidea (a non-ditrysian superfamily). All of these superfamilies are shown to be monophyletic with the exception of the Papilionoidea. According to the recent consensus view of lepidopteran relationships by Kristensen and Skalski [16] (Fig 7A), the Bombycoidea, Geometroidea, Noctuoidea, and Papilionoidea are a group of the Macrolepidoptera; the Pyraloidea, together with the Macrolepidoptera are considered as the Obtectonera; the Tortricoidea together with the Obtectonera are considered as Apoditrysia. However, in our analysis, the superfamily Pyraloidea was placed within the Macrolepidoptera instead of the Papilionoidea. The phylogenetic relationships reconstructed in our study showed that Papilionoidea was a sister to the clade of (Pyraloidea+(Noctuoidea+(Bombycoidea+ Geometroidea))), which is congruent with previous studies [14,51,70–72, 74, 79–82], but differs from the morphological analysis of Kristensen and Skalski [16].

The phylogenetic relationships between Papilionoidea and Hesperioidea have been a subject of controversy in a long time [80]. Traditionally, Papilionoidea and Hesperioidea are considered as two different superfamilies. Hesperiidae (skippers) are usually placed in their own superfamily Hesperioidea while all other butterflies (Papilionidae, Pieridae, Lycaenidae and Nymphalidae) are placed in Papilionoidea [83]. Papilionoidea and Hesperioidea were proposed to be sister group based on a total-evidence analysis of both traditional morphological characters and new molecular characters from three gene regions (COXI, EF-1α and *wingless*) [83]. However, our results indicated that the superfamily Hesperioidea was placed within Papilionoidea and shared close relationships with (Pieridae+(Lycaenidae+Nymphalidae)), which is in accordance with previous studies based on morphological characters, nuclear genes, and mitogenome sequences [14,42,77,84].

Pyraloidea is not considered as being in the Macrolepidoptera, but grouped with the Macrolepidoptera, whereas the Papilionoidea is more distantly related from Macrolepidoptera [77]. According to the yet-to-be-tested hypothesis of Regier et al. [77], the position of the thoracic or abdominal ultrasound-detecting ‘‘ears”, which have never been previously theorized to have a common origin, are a candidate synapomorphy supporting Pyraloidea as a member of the Macrolepidoptera. Phylogenetic analysis by Regier et al. [77], based on five protein-coding nuclear genes (6.7 kb in total) of 123 species representing 55 families from 27 superfamilies of Ditrysia yielded the tree topologies were very similar to ours, but our tree topologies were even more strongly supported. Phylogenetic analysis in our study suggests that the complete mitogenome sequences are significant molecular markers for deep-level phylogenetic studies to verify morphological relationships and reconstruct phylogenetic relationships.

## References

[1] Boore JL. Animal mitochondrial genomes. Nucleic Acids Res. 1999; 27: 1767–1780. 10101183 10.1093/nar/27.8.1767 PMC148383

[2] Wolstenholme DR. Animal mitochondrial DNA: structure and evolution. Int Rev Cytol. 1992; 141: 173–216. 1452431 10.1016/s0074-7696(08)62066-5

[3] Shadel GS, Clayton DA. Mitochondrial transcription initiation. Variation and conservation. J Biol Chem. 1993; 268: 16083–16086. 8344889

[4] Taanman JW. The mitochondrial genome: structure, transcription, translation and replication. BBA-Bioenergetics. 1999; 1410: 103–123. 10076021 10.1016/s0005-2728(98)00161-3

[5] Wei SJ, Shi M, He JH, Sharkey M, Chen XX. The complete mitochondrial genome of *Diadegma semiclausum* (Hymenoptera: Ichneumonidae) indicates extensive independent evolutionary events. Genome. 2009; 52: 308–319. 10.1139/g09-008 19370087

[6] Scheffler I. Mitochondria Wiley-Liss, New York; 1999.

[7] Cameron SL, Whiting MF. The complete mitochondrial genome of the tobacco hornworm, *Manduca sexta*, (Insecta: Lepidoptera: Sphingidae), and an examination of mitochondrial gene variability within butterflies and moths. Gene. 2008; 408: 112–123. 18065166 10.1016/j.gene.2007.10.023

[8] Kim I, Cha SY, Yoon MH, Hwang JS, Lee SM, Sohn HD, et al The complete nucleotide sequence and gene organization of the mitochondrial genome of the oriental mole cricket, *Gryllotalpa orientalis* (Orthoptera: Gryllotalpidae). Gene. 2005; 353: 155–168. 15950403 10.1016/j.gene.2005.04.019

[9] Zakharov EV, Caterino MS, Sperling FAH. Molecular phylogeny, historical biogeography, and divergence time estimates for swallowtail butterflies of the genus *Papilio* (Lepidoptera: Papilionidae). Syst Biol. 2004; 53: 193–215. 15205049 10.1080/10635150490423403

[10] Hurst GDD, Jiggins FM. Problems with mitochondrial DNA as a marker in population, phylogeographic and phylogenetic studies: the effects of inherited symbionts. P Roy Soc B-Biol Sci. 2005; 272: 1525–1534. 16048766 10.1098/rspb.2005.3056 PMC1559843

[11] Avise JC, Nelson WS, Bowen BW, Walker D. Phylogeography of colonially nesting seabirds, with special reference to global matrilineal patterns in the sooty tern (*Sterna fuscata*). Mol Ecol. 2000; 9: 1783–1792. 11091314 10.1046/j.1365-294x.2000.01068.x

[12] Cameron SL, Barker SC, Whiting MF. Mitochondrial genomics and the new insect order Mantophasmatodea. Mol Phylogenet Evol 2006; 38: 274–279. 16321547 10.1016/j.ympev.2005.09.020

[13] Cameron SL, Lambkin CL, Barker SC, Whiting MF. A mitochondrial genome phylogeny of Diptera: whole genome sequence data accurately resolve relationships over broad timescales with high precision. Syst Entomol. 2007; 32: 40–59.

[14] Kim MJ, Kang AR, Jeong HC, Kim KG, Kim I. Reconstructing intraordinal relationships in Lepidoptera using mitochondrial genome data with the description of two newly sequenced lycaenids, *Spindasis takanonis* and *Protantigius superans* (Lepidoptera: Lycaenidae). Mol Phylogenet Evol. 2011; 61: 436–445. 10.1016/j.ympev.2011.07.013 21816227

[15] Kristensen NP, Scoble MJ, Karsholt O. Lepidoptera phylogeny and systematics: the state of inventorying moth and butterfly diversity. Zootaxa. 2007; 1668: 699–747.

[16] Kristensen NP, Skalski AW. Phylogeny and paleontology In: Kristensen NP, editor. Lepidoptera: Moths and Butterflies. Evolution, Systematics, and Biogeography, Handbook of Zoology Vol I, Part 35. Berlin and New York: De Gruyter; 1999 pp. 7–25.

[17] Chai HN, Du YZ, Zhai BP. Characterization of the complete mitochondrial genomes of *Cnaphalocrocis medinalis* and *Chilo suppressalis* (Lepidoptera: Pyralidae). Int J Biol Sci. 2012; 8: 561–579. 10.7150/ijbs.3540 22532789 PMC3334671

[18] Li W, Zhang X, Fan Z, Yue B, Huang F, King E, et al Structural characteristics and phylogenetic analysis of the mitochondrial genome of the sugarcane borer, *Diatraea saccharalis* (Lepidoptera: Crambidae). DNA Cell Biol. 2011; 30: 3–8. 10.1089/dna.2010.1058 20849253

[19] Wu QL, Gong YJ, Shi BC, Gu Y, Wei SJ. The complete mitochondrial genome of the yellow peach moth *Dichocrocis punctiferalis* (Lepidoptera: Pyralidae). Mitochondr DNA. 2013; 24: 105–107. 10.3109/19401736.2012.726621 23025509

[20] Coates BS, Sumerford DV, Hellmich RL, Lewis LC. Partial mitochondrial genome sequences of *Ostrinia nubilalis* and *Ostrinia furnicalis* . Int J Biol Sci. 2005; 1: 13–18. 15951845 10.7150/ijbs.1.13 PMC1140353

[21] Cao SS, Yu WW, Sun M, Du YZ. Characterization of the complete mitochondrial genome of *Tryporyza incertulas*, in comparison with seven other Pyralid moths. Gene. 2014; 533: 356–365. 10.1016/j.gene.2013.07.072 23954873

[22] Park JS, Kim MJ, Kim SS, Kim I. Complete mitochondrial genome of an aquatic moth, *Elophila interruptalis* (Lepidoptera: Crambidae). Mitochondr DNA. 2013; 1–3. 10.3109/19401736.2013.800504 23795838

[23] Park J S, Kim M J, Ahn S J, Kim I. Complete mitochondrial genome of the grass moth Glyphodes quadrimaculalis (Lepidoptera: Crambidae). Mitochondr DNA, 2013: 1–3. 10.3109/19401736.2013.823183 24021007

[24] Traut W, Vogel H, Glöckner G, Hartmann E, Heckel DG. High-throughput sequencing of a single chromosome: a moth W chromosome. Chromosome Res. 2013; 21: 491–505. 10.1007/s10577-013-9376-6 23949445

[25] Zou Y, Ma W, Zhang L, He S, Zhang X, Zeng T. The complete mitochondrial genome of the bean pod borer, *Maruca testulalis* (Lepidoptera: Crambidae: Spilomelinae). Mitochondr DNA. 2014; 1–2. 10.3109/19401736.2014.913167 24779595

[26] Ye F, You P. The complete mitochondrial genome of *Paracymoriza distinctalis* (Lepidoptera: Crambidae). Mitochondr DNA. 2014; 1–2. 10.3109/19401736.2013.869678 24438259

[27] Haines WP, Rubinoff D. Molecular phylogenetics of the moth genus *Omiodes Guenee* (Crambidae: Spilomelinae), and the origins of the Hawaiian lineage. Mol Phylogenet Evol. 2012; 65: 305–316. 10.1016/j.ympev.2012.06.021 22772027

[28] Hebert PDN, de Waard JR, Zakharov EV, Prosser SWJ, Sones JE, Mantle B, et al A DNA 'Barcode Blitz': Rapid Digitization and Sequencing of a Natural History Collection. PLoS One. 2013; 8: e68535 10.1371/journal.pone.0068535 23874660 PMC3707885

[29] Sambrook J, Russell D. Molecular cloning: a laboratory manual 3rd ed., New York, Cold Spring Harbor: Cold Spring Harbor Laboratory Press; 2001.

[30] Simon C, Buckley TR, Frati F, Stewart JB, Beckenbach AT. Incorporating molecular evolution into phylogenetic analysis, and a new compilation of conserved polymerase chain reaction primers for animal mitochondrial DNA. Annu Rev Ecol Evol S. 2006; 37: 545–579.

[31] Bybee SM, Taylor SD, Riley Nelson C, Whiting MF. A phylogeny of robber flies (Diptera: Asilidae) at the subfamilial level: molecular evidence. Mol Phylogenet Evol. 2004; 30: 789–797. 15012956 10.1016/S1055-7903(03)00253-7

[32] Skerratt LF, Campbell NJH, Murrell A, Walton S, Kemp D, Barker S. The mitochondrial 12S gene is a suitable marker of populations of *Sarcoptes scabiei* from wombats, dogs and humans in Australia. Parasitol Res. 2002; 88: 376–379. 11999028 10.1007/s00436-001-0556-5

[33] Thompson JD, Gibson TJ, Plewniak F, Jeanmougin F, Higgins DG. The CLUSTALX windows interface: flexible strategies for multiple sequence alignment aided by quality analysis tools. Nucleic Acids Res. 1997; 25: 4876–4882. 9396791 10.1093/nar/25.24.4876 PMC147148

[34] Tamura K, Stecher G, Peterson D, Filipski A, Kumar S. MEGA6: Molecular Evolutionary Genetics Analysis Version 6.0. Mol Biol Evol. 2013; 30: 2725–2729. 10.1093/molbev/mst197 24132122 PMC3840312

[35] Perna NT, Kocher TD. Patterns of nucleotide composition at fourfold degenerate sites of animal mitochondrial genomes. J Mol Evol. 1995; 41: 353–358. 7563121 10.1007/BF00186547

[36] Lowe TM, Eddy SR. tRNAscan-SE: A program for improved detection of transfer RNA genes in genomic sequence. Nucleic Acids Res. 1997; 25: 955–964. 9023104 10.1093/nar/25.5.955 PMC146525

[37] Beard C, Hamm D, Collins F. The mitochondrial genome of the mosquito *Anopheles gambiae*: DNA sequence, genome organization, and comparisons with mitochondrial sequences of other insects. Insect Mol Biol. 1993; 2: 103–124. 9087549 10.1111/j.1365-2583.1993.tb00131.x

[38] de Bruijn MH. *Drosophila melanogaster* mitochondrial DNA, a novel organization and genetic code. Nature. 1983; 304: 234–241. 6408489 10.1038/304234a0

[39] Akaike H. A new look at the statistical model identification. IEEE T Automat Contr. 1974; 19: 716–723.

[40] Posada D, Crandall KA. Modeltest: testing the model of DNA substitution. Bioinformatics. 1998; 14: 817–818. 9918953 10.1093/bioinformatics/14.9.817

[41] Huelsenbeck JP, Ronquist F. MRBAYES: Bayesian inference of phylogenetic trees. Bioinformatics. 2001; 17: 754–755. 11524383 10.1093/bioinformatics/17.8.754

[42] Mutanen M, Wahlberg N, Kaila L. Comprehensive gene and taxon coverage elucidates radiation patterns in moths and butterflies. Proc R Soc B. 2010; 277: 2839–2848. 10.1098/rspb.2010.0392 20444718 PMC2981981

[43] Guindon S, Lethiec F, Duroux P, Gascuel O. PHYML Online—a web server for fast maximum likelihood-based phylogenetic inference. Nucleic Acids Res. 2005; 33: W557–W559. 15980534 10.1093/nar/gki352 PMC1160113

[44] Hillis DM, Bull JJ. An empirical test of bootstrapping as a method for assessing confidence in phylogenetic analysis. Syst Biol. 1993; 42: 182–192.

[45] Rambaut A. FigTree, a graphical viewer of phylogenetic trees. 2007. Available: http://treebioed.ac.uk/software/figtree.

[46] Cao YQ, Ma CA, Chen JY, Yang DR. The complete mitochondrial genomes of two ghost moths, *Thitarodes renzhiensis* and *Thitarodes yunnanensis*: the ancestral gene arrangement in Lepidoptera. BMC Genomics. 2012; 13: 276 10.1186/1471-2164-13-276 22726496 PMC3463433

[47] Boore JL, Lavrov DV, Brown WM. Gene translocation links insects and crustaceans. Nature. 1998; 392: 667–668. 9565028 10.1038/33577

[48] Kim I, Lee EM, Seol KY, Yun EY, Lee YB, Hwang JS, et al The mitochondrial genome of the Korean hairstreak, *Coreana raphaelis* (Lepidoptera: Lycaenidae). Insect Mol Biol. 2006; 15: 217–225. 16640732 10.1111/j.1365-2583.2006.00630.x

[49] Ojala D, Montoya J, Attardi G. tRNA punctuation model of RNA processing in human mitochondria. Nature. 1981; 290: 470–474. 7219536 10.1038/290470a0

[50] Anderson S, Bankier AT, Barrell BG, de Bruijn MH, Coulson AR, Drouin J, et al Sequence and organization of the human mitochondrial genome. Nature. 1981; 290: 457–465. 7219534 10.1038/290457a0

[51] Hao J, Sun Q, Zhao H, Sun X, Gai Y, Yang Q. The Complete Mitochondrial Genome of *Ctenoptilum vasava* (Lepidoptera: Hesperiidae: Pyrginae) and Its Phylogenetic Implication. Comp Funct Genom. 2012; 2012. 10.1155/2012/328049 PMC3335176 22577351

[52] Yamauchi MM, Miya MU, Nishida M. Complete mitochondrial DNA sequence of the Japanese spiny lobster, *Panulirus japonicus* (Crustacea: Decapoda). Gene. 2002; 295: 89–96. 12242015 10.1016/s0378-1119(02)00824-7

[53] Nardi F, Carapelli A, Dallai R, Frati F. The mitochondrial genome of the olive fly *Bactrocera oleae*: two haplotypes from distant geographical locations. Insect Mol Biol. 2003; 12: 605–611. 14986921 10.1046/j.1365-2583.2003.00445.x

[54] Lutz-Bonengel S, Sänger T, Pollak S, Szibor R. Different methods to determine length heteroplasmy within the mitochondrial control region. Int J Legal Med. 2004; 118: 274–281. 15160269 10.1007/s00414-004-0457-0

[55] Ogoh K, Ohmiya Y. Complete mitochondrial DNA sequence of the sea-firefly, *Vargula hilgendorfii* (Crustacea, Ostracoda) with duplicate control regions. Gene. 2004; 327: 131–139. 14960368 10.1016/j.gene.2003.11.011

[56] Yukuhiro K, Sezutsu H, Itoh M, Shimizu K, Banno Y. Significant levels of sequence divergence and gene Rearrangements have occurred between the mitochondrial Genomes of the wild mulberry silkmoth, *Bombyx mandarina*, and its close relative, the domesticated silkmoth, *Bombyx mori* . Mol Biol Evol. 2002; 19: 1385–1389. 12140251 10.1093/oxfordjournals.molbev.a004200

[57] Clary DO, Wolstenholme DR. The mitochondrial DNA molecular of *Drosophila yakuba*: nucleotide sequence, gene organization, and genetic code. J Mol Evol. 1985; 22: 252–271. 3001325 10.1007/BF02099755

[58] Flook PK, Rowell CHF, Gellissen G. The sequence, organization, and evolution of the *Locusta migratoria* mitochondrial genome. J Mol Evol. 1995; 41: 928–941. 8587138 10.1007/BF00173173

[59] Crease TJ. The complete sequence of the mitochondrial genome of *Daphnia pulex* (Cladocera: Crustacea). Gene. 1999; 233: 89–99. 10375625 10.1016/s0378-1119(99)00151-1

[60] Ballard JWO. Comparative genomics of mitochondrial DNA in members of the *Drosophila melanogaster* subgroup. J Mol Evol. 2000; 51: 48–63. 10903372 10.1007/s002390010066

[61] Mitchell SE, Cockburn AF, Seawright JA. The mitochondrial genome of *Anopheles quadrimaculatus* species A: complete nucleotide sequence and gene organization. Genome. 1993; 36: 1058–1073. 8112570 10.1139/g93-141

[62] Spanos L, Koutroumbas G, Kotsyfakis M, Louis C. The mitochondrial genome of the Mediterranean fruit fly, *Ceratitis capitata* . Insect Mol Biol. 2000; 9: 139–144. 10762421 10.1046/j.1365-2583.2000.00165.x

[63] Wilson K, Cahill V, Ballment E, Benzie J. The complete sequence of the mitochondrial genome of the crustacean *Penaeus monodon*: are malacostracan crustaceans more closely related to insects than to branchiopods? Mol Biol Evol. 2000; 17: 863–874. 10833192 10.1093/oxfordjournals.molbev.a026366

[64] Liu Y, Li Y, Pan M, Dai F, Zhu X, Lu C, et al The complete mitochondrial genome of the Chinese oak silkmoth, *Antheraea pernyi* (Lepidoptera: Saturniidae). Acta Bioch Bioph Sin. 2008; 40: 693–703. 18685785

[65] Feng X, Liu DF, Wang NX, Zhu CD, Jiang GF. The mitochondrial genome of the butterfly *Papilio xuthus* (Lepidoptera: Papilionidae) and related phylogenetic analyses. Mol Biol Rep. 2010; 37: 3877–3888. 10.1007/s11033-010-0044-z 20213506

[66] Krzywinski J, Grushko OG, Besansky NJ. Analysis of the complete mitochondrial DNA from *Anopheles funestus*: An improved dipteran mitochondrial genome annotation and a temporal dimension of mosquito evolution. Mol Phylogenet Evol. 2006; 39: 417–423. 16473530 10.1016/j.ympev.2006.01.006

[67] Margam VM, Coates BS, Hellmich RL, Agunbiade T, Seufferheld MJ, Sun W, et al Mitochondrial Genome Sequence and Expression Profiling for the Legume Pod Borer *Maruca vitrata* (Lepidoptera: Crambidae). PLoS One. 2011; 6, e16444 10.1371/journal.pone.0016444 21311752 PMC3032770

[68] Kim MI, Baek JY, Kim MJ, Jeong HC, Kim KG, Bae CH, et al Complete nucleotide sequence and organization of the mitogenome of the red-spotted apollo butterfly, *Parnassius bremeri* (Lepidoptera: Papilionidae) and comparison with other lepidopteran insects. Mol Cells. 2009; 28: 347–363. 10.1007/s10059-009-0129-5 19823774

[69] Lavrov DV, Brown WM, Boore JL. A novel type of RNA editing occurs in the mitochondrial tRNAs of the centipede *Lithobius forficatus* . Proc Natl Acad Sci USA. 2000; 97: 13738–13742. 11095730 10.1073/pnas.250402997 PMC17645

[70] Lu HF, Su TJ, Luo AR, Zhu CD, Wu CS. Characterization of the complete mitochondrion genome of Diurnal Moth *Amata emma* (Butler) (Lepidoptera: Erebidae) and its phylogenetic implications. PLoS One. 2013; 8: e72410 10.1371/journal.pone.0072410 24069145 PMC3771990

[71] Wu YP, Zhao JL, Su TJ, Li J, Yu F, Chesters D, et al The complete mitochondrial genome of *Leucoptera malifoliella* Costa (Lepidoptera: Lyonetiidae). DNA Cell Biol. 2012; 31: 1508–1522. 22856872 10.1089/dna.2012.1642 PMC3458623

[72] Wei SJ, Shi BC, Gong YJ, Li Q, Chen XX. Characterization of the mitochondrial genome of the diamondback moth *Plutella xylostella* (Lepidoptera: Plutellidae) and phylogenetic analysis of advanced moths and butterflies. DNA Cell Biol. 2013; 32: 173–187. 10.1089/dna.2012.1942 23496766

[73] Wei SJ, Min S, Sharkey MJ, Achterberg C, Chen XX. Comparative mitogenomics of Braconidae (Insecta: Hymenoptera) and the phylogenetic utility of mitochondrial genomes with special reference to Holometabolous insects. BMC Genomics. 2010;11: 371 10.1186/1471-2164-11-371 20537196 PMC2890569

[74] Liao F, Wang L, Wu S, Li YP, Zhao L, Huang GM, et al The complete mitochondrial genome of the fall webworm, *Hyphantria cunea* (Lepidoptera: Arctiidae). Int J Biol Sci. 2010; 6: 172–186. 20376208 10.7150/ijbs.6.172 PMC2850540

[75] Saito S, Tamura K, Aotsuka T. Replication origin of mitochondrial DNA in insects. Genetics. 2005; 171: 1695–1705. 16118189 10.1534/genetics.105.046243 PMC1456096

[76] Zhang DX, Hewitt GM. Insect mitochondrial control region: A review of its structure, evolution and usefulness in evolutionary studies. Biochem Syst Ecol. 1997; 25: 99–120.

[77] Regier JC, Zwick A, Cummings MP, Kawahara AY, Cho S, Weller S, et al Toward reconstructing the evolution of advanced moths and butterflies (Lepidoptera: Ditrysia): an initial molecular study. BMC Evol Biol. 2009; 9: 280 10.1186/1471-2148-9-280 19954545 PMC2796670

[78] Nuss M. Global information system of Pyraloidea (GlobIZ). 2006. Available: http://www.pyraloidea.org.

[79] van Nieukerken EJ, Kaila L, Kitching IJ, Kristensen P, Lees DC, Minet J, et al In: Zhang ZQ, editor. Order Lepidoptera. Animal Biodiversity: An Outline of Higher-Level Classification and Survey of Taxonomic Richness New Zealand, Auckland: Magnolia Press; 2011 pp. 212–221.

[80] Minet J. Tentative reconstruction of the ditrysian phylogeny (Lepidoptera: Glossata). Insect Syst Evol. 1991; 22: 69–95.

[81] Jiang ST, Hong GY, Yu M, Li N, Yang Y, Liu YQ, et al Characterization of the complete mitochondrial genome of the giant silkworm moth, *Eriogyna pyretorum* (Lepidoptera: Saturniidae). Int J Biol Sci. 2009; 5: 351 19471586 10.7150/ijbs.5.351 PMC2686093

[82] Yang X, Xue D, Han H. The complete mitochondrial genome of *Biston panterinaria* (Lepidoptera: Geometridae), with phylogenetic utility of mitochondrial genome in the Lepidoptera. Gene. 2013; 515: 349–358. 10.1016/j.gene.2012.11.031 23220020

[83] Wahlberg N, Braby MF, Brower AV, de Jong R, Lee MM, Nylin S, et al Synergistic effects of combining morphological and molecular data in resolving the phylogeny of butterflies and skippers. Proc R Soc B. 2005; 272: 1577–1586. 16048773 10.1098/rspb.2005.3124 PMC1560179

[84] Heikkilä M, Kaila L, Mutanen M, Peña C, Wahlberg N. Cretaceous origin and repeated tertiary diversification of the redefined butterflies. Proc R Soc B. 2012; 279: 1093–1099. 10.1098/rspb.2011.1430 21920981 PMC3267136

